# Recent applications of metabolomics in plant breeding

**DOI:** 10.1270/jsbbs.21065

**Published:** 2022-02-03

**Authors:** Nozomu Sakurai

**Affiliations:** 1 Bioinformation and DDBJ Center, National Institute of Genetics, 1111 Yata, Mishima, Shizuoka 411-8540, Japan

**Keywords:** metabolomics, mass spectrometry, nuclear magnetic resonance, mQTL, mGWAS

## Abstract

Metabolites play a central role in maintaining organismal life and in defining crop phenotypes, such as nutritional value, fragrance, color, and stress resistance. Among the ‘omes’ in biology, the metabolome is the closest to the phenotype. Consequently, metabolomics has been applied to crop improvement as method for monitoring changes in chemical compositions, clarifying the mechanisms underlying cellular functions, discovering markers and diagnostics, and phenotyping for mQTL, mGWAS, and metabolite-genome predictions. In this review, 359 reports of the most recent applications of metabolomics to plant breeding-related studies were examined. In addition to the major crops, more than 160 other crops including rare medicinal plants were considered. One bottleneck associated with using metabolomics is the wide array of instruments that are used to obtain data and the ambiguity associated with metabolite identification and quantification. To further the application of metabolomics to plant breeding, the features and perspectives of the technology are discussed.

## Introduction

The metabolites of agricultural and horticultural crops have a direct effect on their phenotypes through their influence on stress tolerance, inter-organismal interactions, color, taste, nutritional value, and shelf life. Metabolites can generally be divided into two groups; primary metabolites, which are essential for maintaining the fundamental life processes of the organism, and secondary (specialized) metabolites, which contribute to processes that are specialized to each organism. Together, the primary and secondary metabolites constitute the metabolome, which, among the ‘omes’ in biology (i.e., genome, transcriptome, and proteome) is considered to reflect the phenotype most closely (Guijas *et al.* 2018, Patti *et al.* 2012).

Metabolomics—technology for the comprehensive detection of small molecules in the samples (Hall 2006)—have been applied to numerous research fields, including plant science, zoology, clinical science, microbiology, environmental science, and plant breeding. Metabolomics has been used to clarify the chemical diversity between different crops, analyze stress response mechanisms, discover biomarkers for recognizing different genotypes and phenotypes, and assess material quality. Integrating metabolome data into genome and transcriptome data can be used to identify genes involved in the biosynthesis or degradation of specific metabolites and quantitative trait loci (mQTL), and to perform metabolic genome-wide association studies (mGWAS). In recent years, the data obtained from these studies have been used for predicting phenotypes (metabolite genomic selection/prediction).

This review examined recent publications on the application of metabolomics to plant breeding. In most of these studies, a variety of analytical platforms were used, primarily because of a lack of a single technology that can detect and quantify the full spectrum of metabolites, i.e., the complete metabolome, produced by plants. Consequently, depending on the purpose of the study, researchers have to select the most optimal platforms by considering the types of chemicals to be analyzed, the required quantification accuracy, and the availability of the platforms. Therefore, the second part of this review provides and overview of the technical features of metabolome analysis with a brief introduction of the instruments and relevant technical points. Finally, future perspectives for the further application of metabolomics in this field are discussed. It is hoped that this information can be used as a guide to further the application of metabolomics in plant breeding.

Numerous excellent reviews on topics that were not covered in great depth in this review have been published recently; for example: sample preparation and data analysis for association mapping (Alseekh and Fernie 2021); mechanism analysis and phenotype predictions (Fernandez *et al.* 2021); integration with other omics data (Scossa *et al.* 2021); a comprehensive review of relevant publications (Razzaq *et al.* 2019, Sharma *et al.* 2021); safety assessment of genetically modified organisms (Bedair and Glenn 2020); elucidating and planning plant domestication (Alseekh *et al.* 2021, Fernie and Yan 2019); and computational tools (Misra 2021). The reader is urged to refer to these studies in detail.

## Application of metabolomics to plant breeding

A literature search of all studies published in the last two years (from January 2019 to July 2021) on the application of metabolomics to plant breeding (described later in detail) produced a total of 359 articles excluding reviews (Supplemental Table 1). The table also contains information on target plant, experimental factor, purpose of the study, instrument used, and links to the original paper to facilitate access to the article.

The literature search was conducted using PubMed, PubMed Central (Sayers *et al.* 2021) and European PubMed Central (Ferguson *et al.* 2021). All of the publications containing two metabolomics-related terms (i.e., metabolome or metabolomics) and ten plant breeding-related terms (i.e., breeding, crop improvement, quantitative trait loci, genome-wide association study, genomic selection, genomic prediction, metabolomic selection, phenotyping, marker, or biomarker) were searched for, with consideration given to inflection of the terms. Redundant results, and results containing animal- and clinical-specific terms, such as “cerebrospinal” were omitted, which gave 601 articles. The title, abstract, and original paper (available for 391 articles) were then checked manually, which left 359 original research articles in which metabolomics was applied to plant breeding. Articles indirectly related to plant breeding, such as studies for biological mechanisms using model plants and marker discovery for the geographical origin of traditional Chinese medicine, are included for use case purposes. Studies that did not use either a mass spectrometer (MS) or a nuclear magnetic resonance (NMR) spectrometer for chemical detection were omitted from the analysis.

### Target plants

Metabolomics was applied to a wide variety of plants (Table 1). The 359 articles that were selected examined 160 plants or plant (sample) categories (hereafter referred to as “classes”); of these classes, only seven classes of major crops (including rice, tomato, maize, wheat, and tea tree) were reported in more than 10 publications. Eighteen plant classes were reported in more than 1% of articles (four publications), and 48% of all articles were related to these 18 plant classes. The remainder of the articles (52%) were related to 132 other classes, and 109 of those classes were reported in a single article. A rare medicinal plant *Ferula asafoetida* indigenous to Kashmir, Afghanistan, and Iran was included in the last criterion (Amini *et al.* 2019). As exemplified by coriander (Song *et al.* 2020) and *Medicago polymorpha* (Cui *et al.* 2021), metabolic profiling was conducted in reports on the genome sequences of these species.

### Purposes of the studies

Of the 359 articles, 166 (46%) analyzed metabolic mechanisms, 117 (33%) examined metabolic profiling, 58 (16%) examined marker discovery or discrimination, 18 (5%) examined mGWAS, 14 (4%) examined mQTL, 9 (3%) examined genomic/metabolic selection/prediction, and 25 (7%) examined other purposes. Many of the papers on mechanism analysis focused on the resistance mechanisms of biotic (insects and pathogens) and abiotic (salinity, temperature, and drought) stresses. The metabolic profiling studies included preliminary and descriptive findings for further detailed mechanism analysis and marker discovery. The genomic/metabolomic prediction studies were conducted on major crops, such as rice, oat, barley, and wheat, as well as on less well known crops, such as blueberry (Ferrão *et al.* 2021). In addition, mGWAS was applied to hops (Feiner *et al.* 2021) and coffee (Gamboa-Becerra *et al.* 2019), and mQTL was applied to *Brachypodium distachyon* (Onda *et al.* 2019) and blackcurrant (Abreu *et al.* 2020).

In an important study on the mechanism of stress response, Wang *et al.* (2021) identified the fluorescence in blue light (BrFLU) gene in Pak Choi; the gene was shown to be related to cold acclimation in a study that combined transcriptomics methods.

As an example of metabolic profiling, Qing *et al.* (2021) demonstrated the absence of genes and metabolites in the biosynthesis of colchicine, which is concerned about a factor for the limited market of *Hemerocallis citrina* Borani. Yu *et al.* (2020) elucidated metabolic profiles of 136 accessions of tea trees as part of a study on characterizing tea genetic resources in China. In a study on marker discovery, Lemaitre-Guillier *et al.* (2021) demonstrated that the profiles of volatile organic compounds (VOCs) can be used as diagnostic markers for stress in grapevines.

Metabolomics was also used to depict the metabolic changes during the breeding and domestication processes of the crops. Schouten *et al.* (2019) conducted metabolic profiling of tomato cultivars in the Netherlands and concluded that increased diversity in the composition of aromatic volatiles from the 1990s onwards might reflect the efforts of breeders trying to improve fruit quality. Although not included in Supplemental Table 1, the use of metabolomics to clarify the domestication process has been reported in tomato (Zhu *et al.* 2018) and wheat (Batyrshina *et al.* 2020, Beleggia *et al.* 2016).

The use of metabolomics as an alternative to assessing the phenotype of a plant using organoleptic tests, which are difficult to standardize and perform at scale, was described by Ferrão *et al.* (2021). The relative scarcity of MS- and NMR-based metabolomics studies for large-scale phenotyping using other methods, such as GWAS and genomic predictions, is likely due to the lack of portable instruments. As a simple and portable phenotyping tool, near-infrared (NIR) spectrometry was used recently (Alves Filho *et al.* 2019, Lane *et al.* 2020, Rincent *et al.* 2018).

The stability of metabolome between crops grown under different conditions is often argued when the metabolomic data are subjected to statistical analysis. Chevalier *et al.* (2021) evaluated phenotypic plasticity in carrot varieties and concluded that a balance between constitutive content and the environmental sensitivity of key metabolites should be considered for quality improvement in carrot and other vegetables. For the assessments of the unintended effects of genetically modified crops, reports have been published on maize varieties (Liu *et al.* 2021) and tomato rootstock (Kodama *et al.* 2021).

### Instruments and equipment used in metabolomics studies

In the 359 reviewed articles, liquid chromatography (LC)-mass spectrometry (MS) was the most commonly used instrument (239 articles, 67%), followed by gas chromatography (GC)-MS (104, 29%), nuclear magnetic resonance (NMR) spectrometry (22, 6%), capillary electrophoresis (CE)-MS (2, 1%), and others (19, 5%). LC-MS was often used using established methods to detect polyphenols such as flavonoids related to antioxidant activity and color production. GC-MS was used to obtain an overview of the metabolites produced by primary metabolism (sugars, amino acids, fatty acids, and organic acids), and also to detect flavor compounds. In numerous studies, multi-instrument platforms are used. Moing *et al.* (2020) used the widest variety of methods including LC-MS, GC-MS, NMR, and flow injection-MS (see section “Overview of metabolomics technology”).

## Overview of metabolomics technology

**Fig. 1.**
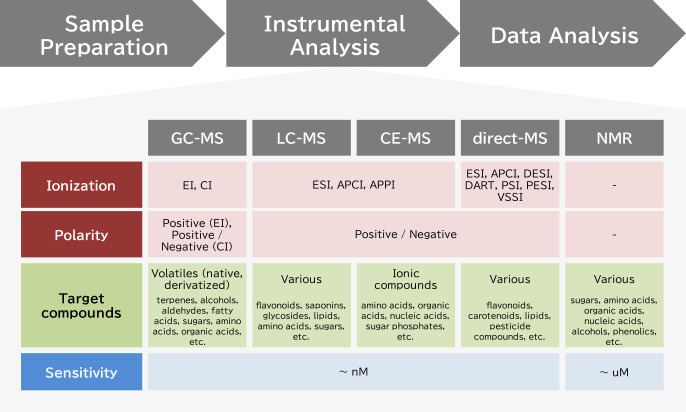
Instruments used for metabolome analysis.

Compared to transcriptome and proteome analysis, the characteristics of metabolome analysis are as follows: 1) a large variety of analytical instruments is used (Fig. 1), primarily because no single method is capable of covering the wide range of physicochemical properties and concentrations of metabolites typically found in samples (Saito and Matsuda 2010). 2) In terms of identifying metabolites, there is often some ambiguity in the obtained results (Viant *et al.* 2017). This is because a comparison of metabolite signals with those from the authentic standard chemical using the same instrumental conditions is required for metabolite identification. Therefore, the metabolomics data may contain known-, predicted- and unknown metabolites. A clear indication of the certainty of the annotation (level 1 to 4) in the results is strongly recommended by the Metabolomics Standards Initiative (MSI) (Sumner *et al.* 2007). 3) There is typically a difference in quantitative accuracy between the data. The quantitative accuracy of metabolite measurements depends on a variety of factors, such as the concentrations in the sample, pretreatment (extraction and concentration), analytical methods, and data processing methods. As a result of these intrinsic features of the technology, metabolome analysis can generally be divided into two categories: targeted analysis, in which specific metabolites are sought with high quantitative accuracy, and untargeted analysis, which tries to comprehensively detect undefined metabolites including unknowns (Gertsman and Barshop 2018). Combinations of targeted and untargeted metabolome analysis and other methods that are used to measure specific compounds can be used in a study. Therefore, selecting the most appropriate analytical method depending on the purpose of the study is necessary for robust metabolome analysis.

This section briefly describes the features of the instruments and some technical considerations for metabolomics experiments, such as sampling and data analysis. Although lipids are one of the largest chemical groups in organisms, this section omits the details of lipid-focused metabolomics (lipidomics). Lipids have multiple functions, and are involved in cellular compartmentation, energy storage, and signaling. The reader is urged to refer to the review by Zullig *et al.* (2020).

### Analytical instruments

It is estimated that more than 1 million metabolites are produced in the plant kingdom (Afendi *et al.* 2012). Two of the most commonly used instruments in metabolomics, mainly because of the wide range of chemicals that can be assayed, are the mass spectrometer (MS), which measures the weight of the ionized molecule, and the nuclear magnetic resonance (NMR) spectrometer, which identifies features of the chemical structure of the molecule being assayed. As the mass measurement in MS is based on the motility of charged ions, an ionization unit (ion source) is placed in front of the MS. Several ionization methods are used. To separate the isomers and isobars (molecules sharing the same mass number) which cannot be distinguished based on mass values, a separation apparatus is coupled to the MS apparatus; for example, LC-MS, GC-MS, and CE-MS are often used.

#### Liquid chromatography-mass spectrometry (LC-MS) 

A wide variety of metabolites can be separated and detected by selecting different column types and solvent conditions. Reversed-phase chromatography with a C_18_ column is often used to detect secondary metabolites with a low to moderate polarity, such as flavonoids, saponins, their glycosides, and polar lipids. Hydrophilic interaction chromatography (HILIC) is also used to better separate amino acids, sugars, and lipid classes. Electrospray ionization (ESI) is the most commonly used, as are atmospheric pressure chemical ionization (APCI) (Hao *et al.* 2020) and atmospheric pressure photoionization (APPI). By changing the voltage polarity, the molecules in a sample can be ionized and detected as their cation (in the positive mode) or anion (in the negative mode).

In addition to measuring the mass of the intact ionized molecule, the mass spectrum of the fragmented ion can be obtained using a modern LC-MS. Indeed, not only can 1) the ion mass be measured, but the mass spectrometer can also be used to 2) select ions with a specific mass value, and 3) fragment the molecular ions via collisions with an inactive gas (typically nitrogen) in a process referred to as collision-induced dissociation (CID). By combining these three functions of MS, the mass spectra of the fragmented ions of the selected precursor ion can be measured. The triple quadrupole-type MS (QqQ and Triple-Q) comprises three MS systems that are connected in tandem, where an ion is selected by the first MS, fragmented in the second MS, and the mass spectra are recorded in the third MS (MS/MS analysis). The Q-ToF-MS and Q-Exactive^®^ have high-resolution MS, namely, time-of-flight (ToF)-MS and Orbitrap^®^-MS, respectively, as the third MS. The ion trap (IT)-type MS can repeat multiple cycles of the selection, fragmentation, and measurement processes (multiple-stage MS analysis). An apparatus combining IT-MS with high-resolution MS (IT-ToF-MS and Fusion Tribrid^®^) is also available. The mass spectrum provides information for the identification, prediction, and discrimination of the metabolites. Fragmentation is also used to quantify the metabolites with high selectivity and sensitivity by selecting the specific precursor ion and measuring the metabolite-specific fragment ion. These approaches are referred to as selected reaction monitoring (SRM), multiple reaction monitoring (MRM), and parallel reaction monitoring (PRM).

LC-MS can be used to measure a wide range of chemicals, as mentioned above, but the variability of the experimental conditions often makes it difficult to compare the results across studies. In addition, special procedures such as the addition of stable isotope-labeled internal controls is required for highly accurate quantification of the metabolites in a crude sample, because ionization rate can be affected by co-existing molecules that are ionized preferentially. This phenomenon is called “ion suppression” or “matrix effect”.

#### Gas chromatography-mass spectrometry (GC-MS) 

GC-MS is widely used for the analysis of volatiles, such as terpenes, alcohols, aldehydes, and fatty acid esters. The metabolites that can be converted to volatile compounds by silylation and esterification (sugars, amino acids, organic acids, and fatty acids) can also be detected (Beale *et al.* 2018). An electron ionization (EI) setting of 70 eV is typically used. Under this condition, the molecule is fragmented during ionization and the mass spectrum is measured as the primary information of the metabolite signal. The fragmentation that occurs due to EI differs from that which occurs in CID (see section on LC-MS) and is more reproducible. Furthermore, the retention time of the metabolites can be standardized by indexing them based on the retention times of alkanes or fatty acid methyl esters. Taken together, these features make it easy to compare the data between studies. Numerous mass spectral libraries are available for compound identification and annotation, including the mass spectral library provided by National Institute of Standards and Technology (NIST, https://chemdata.nist.gov/), Wiley Registry of Mass Spectral Data (Wiley & Sons Inc.), the Golm Metabolome Database (Kopka *et al.* 2005), the library developed by FihenLib (Kind *et al.* 2009), FFNSC 3 for fragrances (Mondello 2015), as well as libraries for pesticides, pollutants, and forensic purposes. As an alternative to MS, a flame ionization detector (FID) is often used. Three studies that used an FID are given in Supplemental Table 1 (Dar *et al.* 2020, Erzen *et al.* 2021, Klevorn *et al.* 2019).

#### Capillary electrophoresis-mass spectrometry (CE-MS) 

CE-MS, which was first applied to metabolomics by Soga *et al.* (2003), is well suited for the detection of hydrophilic ionic metabolites, such as amino acids, organic acids, nucleic acids, and sugar phosphates, i.e., most of the primary metabolites in cells (Buko 2017, Zhang and Ramautar 2021). While ESI is usually used for this purpose, APCI and APPI are also applicable (Hommerson *et al.* 2009a, 2009b).

#### Nuclear magnetic resonance (NMR) spectroscopy 

NMR is advantageous for high throughput targeted analysis because the sample is measured in an enclosed glass vial which prevents contamination of the detector. In addition, sample preparation is straightforward as there is no need for extraction and pretreatment, and samples can be recovered after measurements and used for other experimental applications. Although ^1^H-NMR is generally used for targeted analysis, NMR can also be used for untargeted analyses. The chemical structure of unknown metabolites can be directly elucidated or annotated by two-dimensional NMR, which is a very important advantage of NMR over MS (Emwas *et al.* 2019, Schripsema 2010). However, NMR requires a higher concentration of target metabolite (~μM), which is disadvantageous when compared to MS (~nM). Nonetheless, quantitative accuracy is high (Pinu *et al.* 2019, Razzaq *et al.* 2019).

### Other methods

#### Direct injection methods 

Samples can be ionized directly and MS measurements can be performed without separating them by GC, LC, or CE. The “direct infusion” method typically uses a single syringe pump for injection of the sample. The “flow injection” method uses an autosampler and the pump systems of the LC without a column. The advantage of these direct methods is the short time required for analysis which makes this method well suited to high throughput analysis. The major disadvantages include the lower sensitivity, difficulties with elucidating the mass spectral data obtained from the mixture of metabolites in the samples, and the ion suppression unavoidable for the mixture. To overcome these issues, a spectral stitching method in which each mass scan is first separated into smaller mass ranges, which are then stitched together (Sarvin *et al.* 2020, Southam *et al.* 2016). Three examples of the use of direct injection-MS are provided in Supplemental Table 1: tomato fertilized with different nitrogen sources (Garcia-Casarrubias *et al.* 2019), metabolic profiling of melon (Moing *et al.* 2020), and polyphenol analysis of sorghum (Hodges *et al.* 2021)

#### Ambient ionization 

Ambient ionization is a method that is used for direct ionization of the chemicals on the surface of an object under atmospheric pressure, such as chemicals on the leaves of a plant (Feider *et al.* 2019). Numerous methods based on a variety of principles have been reported to date, including solvent-based desorption electrospray ionization (DESI) and plasma-based direct analysis in real-time (DART) which is based on the same principle as APCI. These ionization techniques are advantageous for real-time monitoring of chemicals on a small area of an object’s surface without pretreatment. These features of ambient ionization methods make them well suited to mass spectrometry imaging. The reader is urged to refer to other reviews on mass spectrometry imaging (Ferguson *et al.* 2019, Hu *et al.* 2021) as a description of these methods is beyond the scope of this review.

#### Ion mobility 

This technique is well suited for high-throughput identification or discrimination of isomers or isobars which can be separated based on their molecular shape (collision cross section, CCS). Numerous MS devices are equipped with an ion mobility unit and CCS libraries of metabolites have recently been constructed (Dodds and Baker 2019, Zhou *et al.* 2020).

#### Supercritical fluid chromatography (SFC) 

SFC is a separation technique that uses supercritical fluids with high diffusivity and low viscosity (Bamba *et al.* 2008, Gordillo 2021, van de Velde *et al.* 2020). These features enable a high-speed and high-resolution separation using a long column. SFC is considered to be a prospective method for the comprehensive analysis of lipids.

### Sample preparation

#### Sampling 

Given the lack of portable instruments for MS- or NMR-based metabolomics analysis in the field, destructive sampling methods are usually necessary. In the case of NMR, non-destructive sampling can be performed if individuals can fit into sample vials. The amount of sample required for the analysis depends on the concentration of the target metabolites, pretreatment methods, and analytical methods, but it typically ranges from 10 to 100 mg fresh weight. Since the metabolome is sensitive to environmental factors, for large-scale sampling such as that required for mGWAS studies, controls for uniform sampling in a short time are required (Gemmer *et al.* 2020, 2021, Song *et al.* 2021, Yao *et al.* 2021). Immediately after sampling, a quenching procedure such as freezing in liquid nitrogen and extraction with organic solvents should be performed in order to avoid chemical changes by enzymatic and chemical reactions.

#### Metabolite extraction 

Metabolite extraction should be performed appropriately for the metabolites targeted for the analysis. The major purpose of extraction is to improve the sensitivity of the analysis by concentrating the targeted metabolites and excluding contaminants, to protect instruments, to stop enzymatic reactions, and to remove proteins. Frequently used methods include the following: solid-phase microextraction (SPME) for volatiles (GC-MS); using a mixture of water, methanol, and chloroform for polar/lipid phase separation, including the Bligh and Dyer protocol and the Folch protocol (GC-MS, lipidome); and using 70–80% methanol for untargeted analyses (LC-MS). In sample preparation for ^1^H-NMR, extraction can be omitted, but dilution with deuterated solvents is necessary in order to avoid absorption by the ^1^H-containing solvents and to stabilize the magnetic field.

#### Derivatization 

Based on the purpose or limitations of the study, derivatization of metabolites is conducted. The major reasons for performing derivatization are to improve the sensitivity of the analysis, to detect metabolites with specific functional groups, to protect instruments, to alter the preference of the detection instruments for specific moieties, and to improve the accuracy of quantification by stable isotope labeling. As mentioned in the section on GC-MS, less volatile compounds such as sugars, amino acids, organic acids, and fatty acids become detectable using GC-MS by converting them to volatile derivatives by silylation and esterification (Beale *et al.* 2018). GC-MS has been used for comprehensive detection of steroids. The derivatization of them to picolinyl esters facilitates the high sensitivity detection of steroids using standard LC-MS conditions with ESI (Honda *et al.* 2010).

#### Controls 

Performing appropriate controls is essential for eliminating false positives, especially for high-sensitivity detection of metabolites by MS. Blank samples, which are prepared excluding the addition of the samples and using the same procedures that are used to prepare the experimental samples, are used to subtract false-positive peaks which can be attributed to contaminants in the solvents and solvent-leaching compounds from plastic tubes and pipette tips. The internal standards (IS) added to the samples at fixed concentration are used to check for, and correct, the reproducibility of retention times and detector sensitivity. Precise checking and correction of the reproducibility and sensitivity can be performed by performing quality control (QC) of samples that contain a larger variety of metabolites. The QC samples are typically prepared by mixing an equal amount of all samples for testing.

### Data analysis

Before analyzing and mining metabolomic data using computational methods such as statistical multivariate analyses, the raw data generated by the MS- and NMR-based instruments should be properly processed and compiled into a data matrix that contains the metabolite signal intensities (concentrations) of each sample to be compared. This preprocessing procedure is comprised of several steps: 1) peak detection and characterization; 2) peak alignment between the samples; and 3) peak identification or annotation.

In GC-MS analysis, the peak signal is represented as a mass spectrum (see the section on GC-MS). When fragment ions with the same mass values are shared among metabolites that are eluted at adjacent retention times, the signals of each fragment ion needs to be assigned appropriately to each metabolite. This process is called deconvolution and is included in step 1.

In LC-MS analysis, the mass values of the ions of intact molecules are detected in step 1. Here, discrimination of the type of the ion has to be performed to estimate the mass values of the original (usually uncharged) molecule. During the ESI process, the neutral molecules are ionized with ions in the peripheral environment (mainly from solvents) to produce adduct ions, such as protonated/deprotonated molecules ([M + H]^+^/[M – H]^–^) and sodium cationized molecules ([M + Na]^+^). Discrimination of the adduct ions is based on the mass differences between the co-eluting mass peaks in the chromatogram. Using the same calculation, identification of the ^13^C-derived stable isotopic peaks, multiply charged ion peaks, and peaks derived from in-source fragmentation in the MS needs to be performed. The proper assignment of MS/MS or a multi-stage MS spectrum to the precursor ion peak is performed in step 1.

Before or after constructing the data matrix by the peak alignment in step 2, normalization of the signals by QC or use of ISs, noise filtering based on the technical replicates and blank filtering are performed. Then the data calculated in step 1 are integrated into the data matrix and used for peak identification and annotation in step 3. The metabolite peaks are identified if the peak of the authentic standard is present and aligned at the same metabolite on the matrix. If authentic standards are not available, the peaks are annotated by searching known metabolite databases and mass spectral libraries, and the structure of the underlying metabolites can then be predicted using the calculated deionized mass values and mass spectra; numerous databases, libraries, and predictive software tools have been developed for this purpose (Misra 2021, O’Shea and Misra 2020).

The same statistical analysis and data mining procedures that are used for the other omics analysis can then be applied using the resulting data matrix.

## Future perspectives

Metabolomic analysis will be applied to the breeding and improvement of a much larger variety of crops in future. In addition to the major crops, minor crops are an important target for future food security (Tadele 2019). For the breeding of roots, tubers, and bananas (RTB), for which the underlying genetic mechanisms are generally poorly understood, large-scale metabolome data have been obtained and a chemotype core collection has been developed (Price *et al.* 2020). It has been reported that integrating the analysis of metabolome data with genomic data increases the predictability of crop traits, such as estimating lipid composition and yields (Campbell *et al.* 2021, Wang *et al.* 2019, Xu *et al.* 2021). However, it has also been suggested that the integration and standalone use of metabolome data does not increase the predictability of yield and plant height, respectively (Gemmer *et al.* 2020, Thorwarth *et al.* 2019). This discrepancy can partly be attributed to the fact that the plant metabolome is markedly affected by environmental factors. In order to more precisely predict complex and environmentally dependent traits, such as yield, further improvements in the selection of an appropriate set of metabolome data for each crop, development of species-specific bioinformatics techniques, and publication of an easy-to-use dataset for bioinformatics researchers are desired.

Improvements in accessibility to metabolomics technologies should also be considered to extend the range of applications of metabolomics to plant breeding because, as stated in this review, there are large variety of the instruments and their data quality. The establishment of networks between metabolomics laboratories and sharing information with plant breeders in each country should also be accelerated. Establishing a metabolomics center that can accommodate a variety of analytical requests, Wuhan MetWare Biotechnology Co., Ltd. in China as an example, is another solution.

Given the increasing demand for high-throughput analyses in mGWAS and prediction studies, the development of rapid and simple techniques is also required. The probe electrospray ionization (PESI) method—an ambient ionization method—is one such prospective technology. Using PESI-MS, the metabolites in a small amount of sample which is collected discontinuously using a probe can be measured rapidly. The advantage of the PESI method is the moderation of ion suppression by sequential and exhaustive ionization (Hiraoka *et al.* 2020, Mandal *et al.* 2011). In addition, vibrating sharp-edge spray ionization (VSSI), which does not require a high voltage, was recently proposed as a compact and inexpensive ionization method (Li *et al.* 2021). Beck *et al.* (2015) described a portable GC-MS for VOC detection in the field. Such technological developments for minimizing the size of detectors are desired. In addition to the methods for precisely detecting individual metabolites described above, improvements in the practical application of portable phenotyping devices is also necessary. For example, the application of near-infrared spectroscopy (NIR) for phenotyping has been recently reported (Alves Filho *et al.* 2019, Lane *et al.* 2020, Rincent *et al.* 2018). The use of chemical sensors, such as electronic noses, is also being investigated (Cui *et al.* 2018, Tholl *et al.* 2021). The development of portable, non-destructive, and inexpensive, but robust chemical detectors, combined with efficient methods reliably correlate device signals to accurate metabolite data will be a major breakthrough in this research area.

The identification of unknown peaks detected by MS- and NMR-based metabolomics is a necessary step for elucidating the underlying mechanisms of the phenotypes. In future breeding studies, crops harboring a wider range of specialized metabolites that characterize the crop phenotypes will be targeted. Among the large number of unknown peaks, such specialized metabolites should be prioritized for further detailed analysis, such as identification, annotation, and examination of functionality. In order to such prioritization, metabolome databases which facilitate comparisons among a wider range of species is needed. An example of a database is the Food Metabolome Repository (http://metabolites.in/foods/) (Sakurai and Shibata 2017), in which untargeted LC-MS data are compared among 222 foodstuffs, including more than 96 raw materials derived from plants. The expansion of such a concept is required in future studies.

## Author Contribution Statement

NS prepared the manuscript.

## Supplementary Material

Supplemental Table

## Figures and Tables

**Table 1.** Plants or plant classes used in breeding-related metabolome analysis that were reviewed in this study (published from Jan 2019 to July 2021)

Plant (classes)	# Articles
Rice	24 (7%)
Tomato	22 (6%)
Maize	18 (5%)
Wheat	18 (5%)
Tea	13 (4%)
Brassicaceae except Arabidopsis	10 (3%)
Traditional Chinese medicine	10 (3%)
Poplar	8 (2%)
Soybean	8 (2%)
Grapevine	6 (2%)
Arabidopsis	5 (1%)
Barley	5 (1%)
Cassava	5 (1%)
Oat	5 (1%)
Sorghum	5 (1%)
Apple	4 (1%)
Medicago	4 (1%)
Olive	4 (1%)
Others	185 (52%)
Total	359
